# Songs of Love, Orthoptera-Style

**DOI:** 10.1371/journal.pbio.1001275

**Published:** 2012-02-28

**Authors:** Michael Greenfield

**Affiliations:** IRBI, CNRS UMR 7162, Université François Rabelais de Tours, Tours, France

## Abstract

Michael Greenfield reviews Cricket Radio, Tuning in the Night-Singing Insects

[Fig pbio-1001275-g001]On a visit to Beijing a number of years ago for a meeting, I left the conference center at midday for a bit of unguided exploration and wandered the streets of one of the city's commercial districts. In the midst of a fully paved and built-upon landscape, with not even the smallest garden in evidence, I heard what was unmistakably katydid (bushcricket) song. Summoning what remained of my ability to track high-frequency sound, a talent that had served me well previously in field work, I found the source: several singers, each caged individually and propped in a storefront where they were alternately calling and feeding on small slices of melon. I had, of course, read much about the “cricket culture” that had developed in China over several millennia and seen exhibits of the paraphernalia in art galleries and museums, but here I came face to face with the real thing. In *Cricket Radio*, John Himmelman captures the general appeal of singing insects such as I had witnessed in Beijing, and he offers us some information and ideas on how we can share in an interest that has brought aesthetic pleasure and spiritual solace, as well as a ready connection with the natural world, to many in various cultures [1]. Himmelman reminds us that beyond the butterflies and moths with their visual beauty, there are other groups of insects that can fascinate rather than repel, and his objective is to help us discover one of them.

**Figure pbio-1001275-g001:**
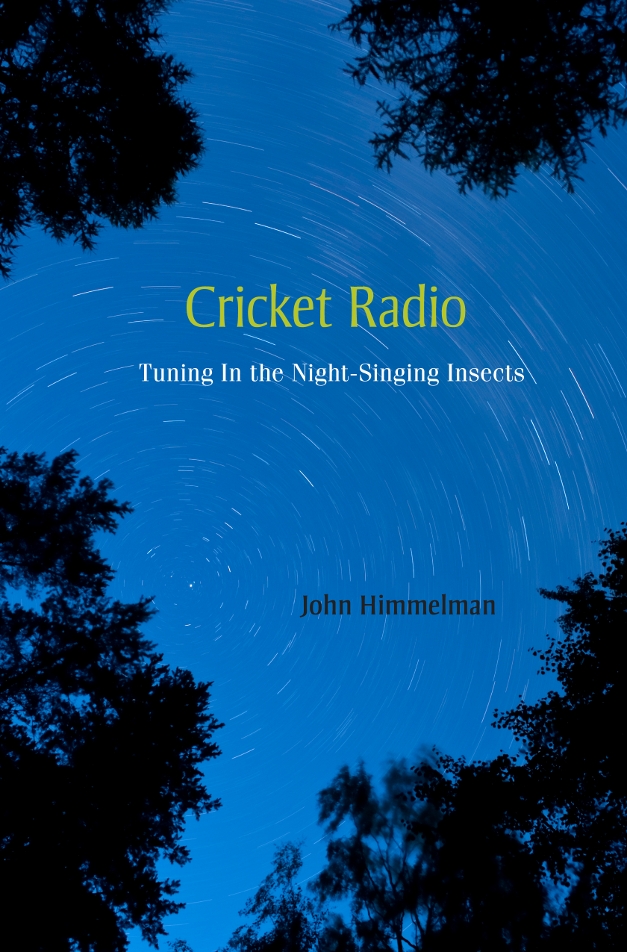
Himmelman J (2011) Cricket Radio: Tuning In the Night-Singing Insects. Cambridge, MA: Harvard University Press. 272 p. ISBN-13: 978-0674046900 (hardcover). US$22.95.

To aid our discovery, Himmelman offers us the basics on how and why these insects sing, includes considerable description of their general natural history—along with the overall classification of crickets and katydids and brief vignettes of the more common species in eastern North America—and introduces some information on the personalities who have been involved with singing insects over the course of history—beginning with antiquity. The writing is straightforward and clear, making the book accessible to all, is developed with personal anecdotes, replete with children's amusements (the author, a lifelong naturalist, has written several scientific books for children), and is largely accurate. I say “largely” because there are a few points where a specialist could quibble, and where a better explanation could have been given while retaining the general clarity of the book.

Studies of acoustic insects have occupied a central role in several topics in evolutionary biology, notably the influences of natural and sexual selection on male and female behaviors during courtship and mating, and the processes by which populations diverge and potentially form separate species. The songs of crickets and katydids are largely signals by which males announce their location, species identity, gender, state of sexual development, and “quality” to females of their species. Some songs may also serve as close-range courtship signals that influence the final mating decisions of females that have arrived in the vicinity of a calling male, as well as declarations of physical strength and “motivation” broadcast toward male rivals. While females in certain species answer male calls with their own “soft songs” in the context of a courtship dialogue, signaling is mostly a male preoccupation. Because loud, incessant calls can attract the attention of predators (e.g., insectivorous bats) and parasites (e.g., parasitoid flies) in addition to females, it has been generally assumed that it is the male who is more risk-prone during mating behavior; here, one might note that this traditional assumption ignores the risks that females moving across the landscape toward a calling male could suffer. Himmelberg duly observes this sexual dichotomy, but he presents it vaguely—and with what amounts to a group selection interpretation—that is, as the “greater importance evolution places on the female when it comes to propagating the species” (p. 13)—rather than focusing on the costs and benefits of reproduction for the individual male. That is, most evolutionary biologists would point out that a male's fitness is often limited by the number of females with whom he mates [2], a feature that can select for a certain degree of risk taking. On the other hand, a female's fitness is influenced more by the quality of her mate and increases relatively little with each additional mate. Thus, more evaluation of mates—and caution during the process—is expected in females, as fewer rewards from risk-prone behavior would be forthcoming.

Among the more intriguing behaviors exhibited by acoustic insects are the ways in which groups of singing males may broadcast a collective chorus characterized by temporally precise interactions between neighbors' songs. In some species these interactions include rather striking orchestral arrangements wherein each singer adjusts his rhythm of regularly repeated calls, with the result that all of the males in the chorus are essentially signaling in phase with one another. Such chorusing is but an example of the general phenomenon of synchrony in nature, occurrences which interest mathematicians and physicists [3] as well as biologists. Himmelberg presents a particularly comprehensive treatment of chorusing, and he focuses specifically on synchrony and why acoustic insects might bother to create it. One hypothesis is that those natural enemies that can menace singing males may not be able to localize any one singer if all synchronize their calls, a cognitive limitation in these enemies. But in covering another hypothesis, Himmelberg falls prey to a fallacy that has beset many scientists over the years, that singing together and at the same time affords the males greater overall sound intensity (higher decibel level, p. 21) and, implicitly, more female-attracting power. The two problems with this argument are that the physics regulating the combination of sounds from multiple sources prevent the overall intensity of all but the largest choruses from being significantly higher than a lone male and that the greater number of females attracted to the chorus would potentially be shared by the males. Consequently, the best that a male in a chorus could do would be to equal the “intensity-based attractiveness” of a lone male, and in most cases he would not even do that. Rather, synchrony may represent a means by which males in a chorus preserve certain elements of the song rhythm that females must hear before responding to any given male, or the phenomenon may simply emerge as a by-product of the simple signal interactions between neighboring males (see p. 22).

Various activities of acoustic insects have entered the North American “natural history lore,” and one of the best known is that of the thermometer cricket, *Oecanthus fultoni*. Because their body temperature remains roughly equivalent to the ambient temperature, most crickets and katydids exhibit a thermal response in which their chirp or pulse rhythm increases as it gets warmer: Their songs are produced by stridulating—rubbing the forewings together—and the contraction rate of the wing muscles is temperature dependent. *O. fultoni* presents a special case because the positive linear relationship between chirp rhythm and temperature is so precise that an observer can determine the temperature by listening to the song: Temperature (°F)  =  (number of chirps broadcast in 13 seconds) + 40. But in describing this phenomenon, one has to clearly define the song units in question. On p. 112 it is noted that the crickets “speed up the frequency of their call,” which is an unfortunate confusion. Frequency normally refers to the number of waves per second in a sound such as a cricket chirp, and this value varies between 3,000 and 7,000 among different cricket species. Unlike chirp rate, the sound frequency within cricket and katydid chirps remains rather constant over a wide temperature range, and the mechanism that allows singing insects to preserve sound frequency while increasing chirp rhythm is an important question in biomechanics.

Readers of *Cricket Radio* should take note that while the book is an excellent introduction to the biology of these animals and how to take delight in them in the field or in one's home, it has certain limits. It applies quite well to the fauna of eastern North America, but readers living west of the Great Plains, or indeed, outside of North America, will find it less useful. Also, as Himmelman emphasizes, his book is not an identification guide, and a novice will not be able to identify species from the verbal descriptions, illustrations, or even information on songs given in the text. Fortunately, readers are referred to the appropriate specialist guides and song CDs. But they should be forewarned that even with these guides, identification of the species that one might stock in his/her “cricket radio” is often quite difficult. Cryptic species—which can only be distinguished following examination by a microscope or via detailed analysis of the male song, and which present some very interesting questions in the study of speciation—abound in several North American genera, and the photographs, either black and white or color, in *Cricket Radio* will not help to discern them. In this regard, I would have preferred more of the line drawings, particularly drawings that illustrate the various points and specific behaviors that Himmelman was writing about. And as for identification, one would really need dichotomous keys [4] and audio oscillograms and spectrograms of the songs. Given the current availability of sound processing software (some of it being freeware) and the ease with which it can be applied with any portable computing device, it would not be unreasonable to expect cricket enthusiasts—novice or veteran—to record a song and compare its graph with that in a key.

From my own perspective as a student of singing insects over the past 30 years, I found *Cricket Radio* to be a good read, and I learned quite a lot from it, particularly certain fine points on behavior, where and how to track down the different species, and tips on keeping them in captivity. Himmelman has evidently paid considerable attention to details of the lives of these insects, and he has made an extraordinary effort to transfer his knowledge and sense of wonder. Whether you are simply interested in the natural history of singing insects or wish to retain them for their accompaniment—musical and otherwise—you will benefit greatly from this book.

**About the Author**

Michael Greenfield is leader of the research group “cognitive ecology” at the CNRS Institute for Insect Biology (IRBI; l'Institut de Recherche sur la Biologie de l'Insecte) and Professeur des Universités at the Université François Rabelais de Tours, France. He received his PhD from the University of Wisconsin for work on species recognition and reproductive isolation in Lepidoptera and held faculty appointments at the University of California, Los Angeles (UCLA, 1981–1991) and the University of Kansas (1991–2005) prior to arriving in Tours. He teaches courses on animal behavior, behavioral ecology, and communication, including human language, and pursues research on sexual selection and animal communication, including the chemical, visual, and acoustic modalities. He has conducted field research on three continents and is the author of *Signalers and Receivers: Mechanisms and Evolution of Arthropod Communication* (2002, Oxford University Press). His current work largely addresses questions on the evolution of sexually selected signal and receiver traits in acoustic insects.
